# Systems-level computational modeling in ischemic stroke: from cells to patients

**DOI:** 10.3389/fphys.2024.1394740

**Published:** 2024-07-02

**Authors:** Geli Li, Yanyong Zhao, Wen Ma, Yuan Gao, Chen Zhao

**Affiliations:** ^1^ Gusu School, Nanjing Medical University, Suzhou, China; ^2^ School of Pharmacy, Nanjing Medical University, Nanjing, China; ^3^ QSPMed Technologies, Nanjing, China; ^4^ The First Affiliated Hospital of Nanjing Medical University, Nanjing, China

**Keywords:** ischemic stroke, system biology, computational modeling, virtual clinical trial, network pharmacology

## Abstract

Ischemic stroke, a significant threat to human life and health, refers to a class of conditions where brain tissue damage is induced following decreased cerebral blood flow. The incidence of ischemic stroke has been steadily increasing globally, and its disease mechanisms are highly complex and involve a multitude of biological mechanisms at various scales from genes all the way to the human body system that can affect the stroke onset, progression, treatment, and prognosis. To complement conventional experimental research methods, computational systems biology modeling can integrate and describe the pathogenic mechanisms of ischemic stroke across multiple biological scales and help identify emergent modulatory principles that drive disease progression and recovery. In addition, by running virtual experiments and trials in computers, these models can efficiently predict and evaluate outcomes of different treatment methods and thereby assist clinical decision-making. In this review, we summarize the current research and application of systems-level computational modeling in the field of ischemic stroke from the multiscale mechanism-based, physics-based and omics-based perspectives and discuss how modeling-driven research frameworks can deliver insights for future stroke research and drug development.

## 1 Introduction

Ischemic stroke is characterized as a cerebral vascular disease that is initially resulting from disruptions in cerebral blood circulation (often due to vascular occlusion) and can ultimately leads to brain tissue damage and cognitive as well as physical impairment (Campbell et al., 2019). Clinically, ischemic stroke manifests primarily as a sudden-onset focal or diffuse neurological deficit which are contributed by numerous etiological factors, exhibiting complex pathophysiological mechanisms across various biological scales. At the cellular and tissue levels, stroke onset and ischemia trigger substantial release of inflammatory cytokines and danger signals from dead cells, which can permeate surrounding tissues and activate inflammatory pathways within cells (Anrather and Iadecola, 2016). This typically involves pivotal receptor-mediated pathways such as TLRs (toll like receptors), TNFRs (tumor necrosis factor receptors), IFNRs (interferon receptors), and various interleukin receptors (Caso et al., 2007; Qin et al., 2022; Xue et al., 2022; Zhu et al., 2022; Kuo et al., 2023). Another essential regulator induced by ischemia is the activation of the HIF (hypoxia-inducible factor) pathway within brain tissues (Luo et al., 2022). The cascade of events downstream of HIF can contribute to angiogenesis, cell survival, and proliferation, thereby playing a vital role in the restoration of damaged brain tissue (Amalia et al., 2020). Ischemia also results in metabolic disturbances in brain cells, hampers the normal functioning of ion pumps which can disrupt intracellular ionic balance and activate pathways leading to cell apoptosis or necrosis (Sekerdag et al., 2018). On the brain microvasculature, the differential regulation of VEGF (vascular endothelial growth factor) pathway is pivotal in regulating angiogenesis, vascular permeability and blood-brain barrier remodeling (Greenberg and Jin, 2013). Following the restoration of blood flow, ischemia-reperfusion injury can occur and further exacerbate the condition due to excessive production of reactive oxygen species (ROS), calcium overload, damage to gap junctions, metabolic dysregulation, and mitochondrial dysfunction (Nour et al., 2013; Al-Mufti et al., 2018; Gauberti et al., 2018). Further at the human body system level, variations in genetics, age, gender, health status, and lifestyle among different patients can result in divergent disease progression and recovery trajectories and this necessitates the investigation of personalized treatment strategies (Zafar et al., 2016). Overall, the complexity of ischemic stroke pathophysiology is at least multi-scale from sophisticated signal transduction and crosstalk at the cellular level, to multi-faceted pathogenic and tissue damage mechanisms at the tissue level, all the way to interindividual variability at the systems level, and this has posed significant challenges to traditional experimental research that aims to identify drug targets and treatment plans given the extremely low success rates of stroke clinical trials in the past two decades (Chen and Wang, 2016; Dhir et al., 2020).

Computational systems biology modeling, grounded in physiology- and data-driven computer simulations, has emerged as a robust and high-throughput approach for interpreting and analyzing complex pathophysiological processes (Kitano, 2002). These computational models have their unique advantages in basic research and drug development as they allow mechanistic integration of multimodal biological knowledge and data across scales and studies (Yue and Dutta, 2022; Zhang et al., 2022), and for complex diseases such as ischemic stroke, the systems-level understandings derived from such models could contribute significantly in translational research. As biological systems are dynamical, ordinary differential equation (ODE) modeling has become one of the most prevalent computational modeling approaches in the field, with a long history originating from the study and simulation of biochemical pathways and gene regulatory networks (Sordo Vieira and Laubenbacher, 2022). A major advantage of ODE modeling is that the ODE model system can be mechanistically constructed and explained given *a priori* knowledge regarding how various system components (e.g., genes, proteins, cells) interact with each other, and the mathematical solution of the ODE system can quantitatively predict time-course profiles of the entire system behavior (including all components) upon particular perturbations which can physiologically mimic disease triggers (Daun et al., 2008). Other modeling approaches besides ODE such as agent-based modeling, logic-based modeling, partial differential equation (PDE) modeling as well as hybrid modeling have also gained popularity in different research fields. PDE modeling, given its strong theoretical basis in physical principles such as mass transfer and reaction-diffusion, are particularly useful in the simulation of biophysical processes such as vascular blood flow (Caiazzo et al., 2018). Thus, considering the disease features of ischemic stroke, ODE and PDE modeling (sometimes used in hybrid) are commonly employed by computational systems biologists to decipher intracellular signal transduction, cell-cell interaction as well as vascular hemodynamics in stroke translational research.

**FIGURE 1:**
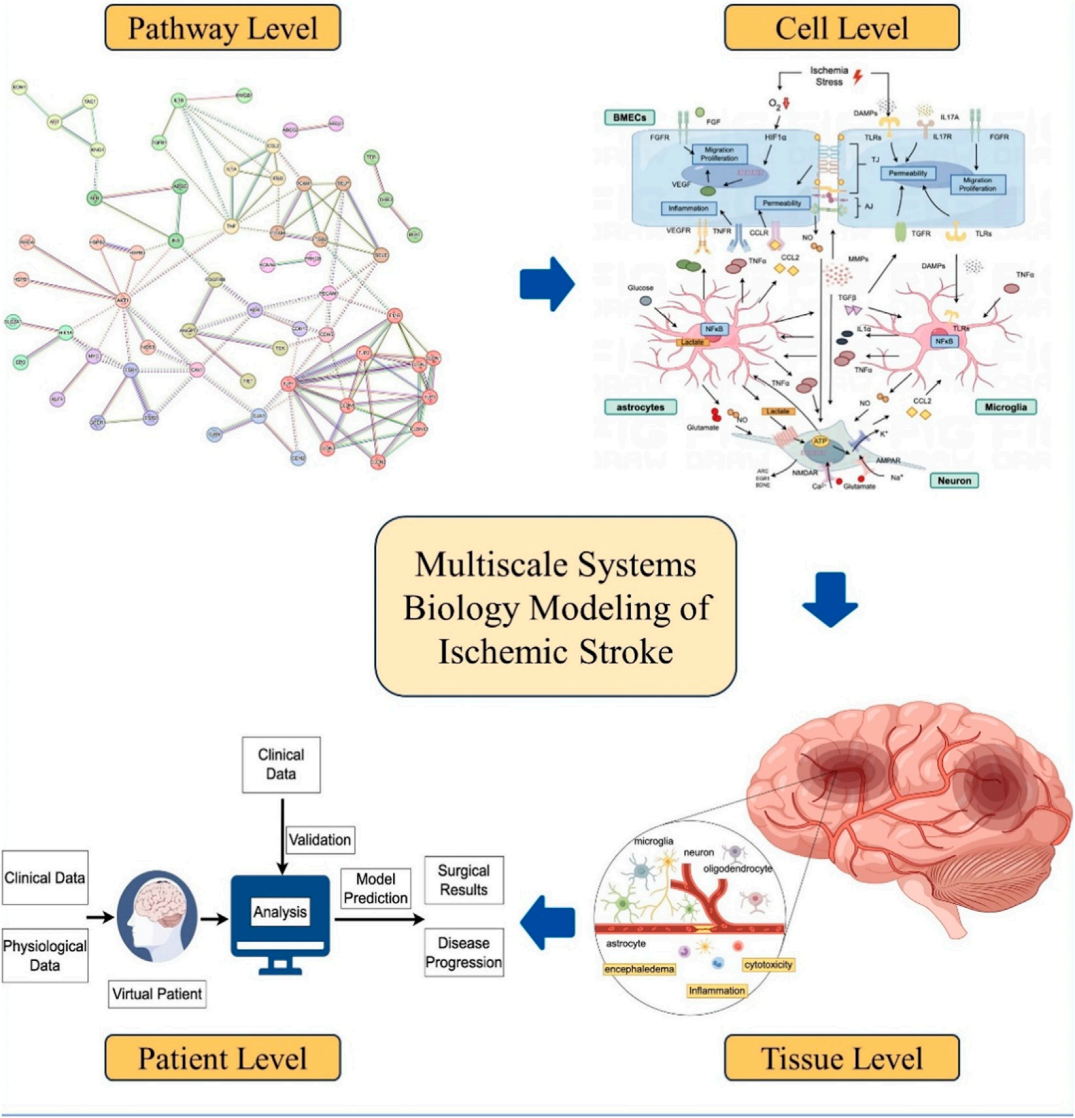
Overview of systems biology modeling at different scales for ischemic stroke translational research (created by Figdraw).

In this review, we explore the application of computational systems biology modeling in ischemic stroke research across various scales, including cellular, tissue, and human body levels (Figure 1). We will also touch upon recent examples that employed multi-omics and network pharmacology approaches for therapeutic target identification and biomarker evaluation in ischemic stroke. We emphasize the role of these modeling analyses in elucidating the complex intracellular signaling patterns in brain cells and the dynamic spatiotemporal changes in brain tissue damage and repair following ischemic stroke, as well as the utilization of computational models to predict individual stroke patient outcome and formulate personalized treatment plans (Table 1; Table 2).

**TABLE 1:** Selected examples of computational systems biology models of ischemic stroke that focus on cell- and tissue-level pathophysiology.

Model scale	Model elements	Model type	Summary of model	Refs
Cell scale	Mitochondria biomolecules and ions	ODE	Described a mathematical model regarding the regulation of intracellular calcium ion, ATP production in ischemic stroke	Diekman et al. (2013)
Cell scale	M1/M2 microglia and cytokines	ODE	Employed systems biology modeling approach to study the regulation and transition of M1/M2 phenotypes of microglia post-stroke	Amato and Arnold (2021)
Cell scale	Intracellular signal pathway proteins and molecules	ODE	Developed a computational model of the brain microvascular endothelial cell to predict molecular targets and drivers of therapeutic response in different CNS diseases	Gorick et al. (2022)
Cell scale	Cytokines and biomolecules	ODE	A mathematical model that included different NVU coupling mechanisms	Sten et al. (2023)
Tissue scale	Ions, metabolites. ion channels	Hybrid ODE-PDE	Computational models of osmosis that included diffusion dynamics of sodium, potassium, calcium, chloride, and bicarbonate ions and the resulting regulation of edema volume following stroke	Dronne et al. (2006), Orlowski et al. (2011), Chander and Chakravarthy (2012), Orlowski et al. (2013)
Tissue scale	Immune cells, neurons and astrocytes	ODE	A computational model that involves the interactions between neurons, astrocytes, microglia, neutrophils, macrophages, cytokines, and chemokines during an ischemic stroke	Di Russo et al. (2010)
Tissue scale	Blood vessel and water filtration	PDE	A model developed to study the effect of reperfusion on the formation of vasogenic oedema during stroke	Mokhtarudin and Payne (2015)
Tissue scale	Brain cells, ions, biomolecules as sub-modules	ODE and PDE	Mechanism-based computational models of brain ischemia, reperfusion injury as well as edema formation during stroke progression	Dronne et al. (2004), Chapuisat et al. (2010)
Tissue scale	Brain cells as units affected by blood flow and oxygen	Algebraic	A 2D model of stroke that incorporates time-dependent evolution of various disease biomarkers including blood flow- and oxygen-related measurement features	Duval et al. (2002)
Tissue scale	Ions, ion channels, transporters, pumps	ODE	A multiscale mechanistic computational model depicting the primary early pathophysiological mechanisms of stroke in rodent and human brains with applications in testing different neuroprotective strategies	Dronne et al. (2008)
Tissue scale	Stem cells and brain cells	ODE	An ODE-based model that examines the influence of stem cell transplantation on the dynamic recovery of brain tissue post-stroke	Alqarni et al. (2021)
Tissue scale	Blood vessel and haematoma	PDE	A 2D mathematical model capable of simulating the effects of hemorrhagic transformation across various scales of vasculature length in stroke	Wang and Payne (2021)

**TABLE 2:** Selected examples of multi-scale clinical trial models for patients with ischemic stroke with focuses on thrombectomy outcome prediction and surgical planning.

Model scale	Model outcome of interest	Model type	Summary of model	Refs
Patient scale	Brain perfusion maps	PDE	A three-compartment porous microcirculation model capable of describing changes in cerebral blood flow perfusion in healthy individuals and ischemic stroke patients	Jozsa et al. (2021)
Patient scale	Thrombectomy procedure and clot status	PDE	Using previously validated methodologies, these models can simulate the thrombectomy procedure in a patient-specific case to identify factors contributing to procedural success or failure	Luraghi et al. (2021a), Benemerito et al. (2023)
Patient scale	Population-level patient functional outcome after thrombectomy	Hybrid	Developed multiscale computational frameworks that can simulate the thrombectomy procedure in patient-specific manners to identify potential factors that contribute to recanalization success or failure	Good et al. (2020), Lopez-Rincon et al. (2020), Miller et al. (2021)

## 2 Modeling response to ischemic stroke at the cellular level

The concept of the Neurovascular Unit (NVU) was introduced in 2001 at a meeting of the National Institute of Neurological Disorders and Stroke as the fundamental unit for studying the mechanisms of interaction between neural and vascular components (Wang et al., 2021; Tiedt et al., 2022). Structurally, the NVU encompasses neurons, glial cells (e.g., astrocytes, microglia), endothelial cells, smooth muscle cells and pericytes. Functionally, the NVU mediates neurovascular coupling (NVC), wherein neurons and astrocytes secrete substances to regulate cerebral blood flow (CBF) (Salinet et al., 2019; Beishon and Minhas, 2021) as well as ensure adequate energy supply and timely clearance of metabolic by-products. NVU also coordinates the formation of the blood-brain barrier (BBB) through the regulation of endothelial cells and pericytes, which is responsible for preventing the entrance of metabolic toxins and safeguarding normal neural functions (Salmina et al., 2021; Naranjo et al., 2022). Following the onset of ischemic stroke, the blood-brain barrier is compromised, resulting in increased permeability which allows metabolic by-products to infiltrate the central nervous system and cause damage to neural cells (Abbott et al., 2010; Prakash and Carmichael, 2015). Subsequently, impaired neural cells exhibit reduced capacity to regulate CBF which results in the accumulation of metabolic waste and accelerates the progression of the disease. Therefore, maintaining the functional integrity of NVU during ischemic stroke is crucial for preserving brain function and mitigating brain injury. Previous studies have demonstrated that mechanistic systems biology modeling can provide a robust framework to describe and analyze complex multiscale biological systems; in the scenario of NVU, this could greatly advance our understanding of the emergent underlying principles that govern the operation of NVU. The subsequent section will discuss mechanism-based computational models specifically developed for the various cellular components of NVU, including astrocytes, microglia, brain microvascular endothelial cells and neurons.

### 2.1 Astrocytes in stroke

Astrocytes, a subtype of glial cells, are integral to the central nervous system (Sofroniew and Vinters, 2010; Endo et al., 2022). They contribute to the structural support surrounding microvessels by secreting extracellular matrix molecules such as proteoglycans and collagens to enhance BBB integrity and maintain a stable environment essential for neuronal function (Linnerbauer et al., 2020; Lee et al., 2022). Astrocytes are interconnected by gap junctions which allow the free exchange of IP3 (inositol 1,4,5-triphosphate) among cells and downstream activation of calcium pathways to promote the release of glutamate, an essential neurotransmitter (Mulica et al., 2021). During the course of ischemic stroke onset and progression, astrocytes can secrete critical neurotrophic and angiogenic factors such as VEGF and BDNF (brain-derived neurotrophic factor) to promote angiogenesis, improve cerebral blood flow, enhance neuronal activity, mitigate inflammatory cascades as well as ischemia-induced tissue damage (Shen et al., 2021; He et al., 2022; Li et al., 2022).

Regarding astrocyte metabolism and function in ischemic stroke, Diekman et al. constructed an ODE-based computational model to describe how changes in IP3-mediated calcium release can affect mitochondrial ATP production during ischemic stroke (Diekman et al., 2013). The model includes a core ATP generation module based on previous work by Magnus and Keizer (1997), and the authors added mechanistic description of calcium ions being absorbed into and pumped out of endoplasmic reticulum, as well as the cellular mechanisms for importing and exporting sodium and potassium ions. Model simulations suggest that, under conditions of hypoxia and glucose deprivation, there is a significant decrease in intracellular ATP concentration that would lead to a decline in Na+/K+ pump activity, membrane depolarization, and cell swelling. Simulations also suggest that activation of P2Y1R (P2Y receptor) is able to reverse these effects, and further analyses suggest that the reversal ability of P2Y1R is associated with IP3-mediated calcium release. The authors then used the model to characterize the timing of reduced mitochondrial ATP production due to decreased levels of acetate or external oxygen as well as the time-course dynamics of how activation of P2Y1R would re-stimulate the ATP production, suggesting a new strategy for astrocyte protection after ischemic stroke. From the model utility standpoint, it is suggested that the model simulations should be further compared with quantitative experimental measurements of cell calcium and ATP concentrations *in vitro*, in order to establish the translational significance of such model-based target identification efforts.

### 2.2 Microglia in stroke

Microglia serve as the brain’s primary line of defense. They respond to tissue inflammation and injury by secreting inflammatory factors as well as phagocytizing damaged cells to maintain immune homeostasis within the neural tissue microenvironment (Colonna and Butovsky, 2017; Lenz and Nelson, 2018). Furthermore, microglia can modulate synaptic formation and remodeling by up- and downregulating the release of neurotransmitters (Zeng et al., 2022). During ischemic stroke, microglia can differentiate into two distinct phenotypes (Ma et al., 2017; Xu et al., 2020). M1-type microglia are known to secrete a variety of pro-inflammatory cytokines including TNFα, IL-1β, and IL-6, which can facilitate the formation of an inflammatory milieu in neural tissue and compromise the integrity of the blood-brain barrier (BBB). M2-type microglia primarily function in an anti-inflammatory and neuroprotective capacity by phagocytizing debris from dead cells; they also release various anti-inflammatory factors such as IL-10 and IL-13 and neurotrophic factors like BDNF and GDNF (glial cell-derived neurotrophic factor) to expedite neural repair (Tang and Le, 2016; Jurga et al., 2020; Guo et al., 2022).

To investigate the complex role of microglia, Alam et al. (2016) explored, from the perspective of intracellular signaling pathways within microglial, the potential therapeutic impact of combining anti-inflammatory pharmacotherapy with transcranial direct current stimulation (tDCS) and repetitive transcranial magnetic stimulation (rTMS) following ischemic stroke using an ODE-based mechanistic model. The authors chose the NFκB (nuclear factor kappa B) signaling pathway to represent the intracellular inflammatory responses, and they also included signaling cascades such as PI3K/AKT, Ca2^+^/Calmodulin (CaM), MAPK (mitogen-activated protein kinase) modules upon the binding of BDNF to its receptor, which is closely related to cellular proliferation and survival. The primary focus was to simulate the effect of post-stroke treatment using the anti-inflammatory drug minocycline in conjunction with tDCS and rTMS. At the molecular level, the model revealed that minocycline can reduce cellular inflammation by inhibiting the activation of NFκB, and moreover the therapeutic application of tDCS and rTMS can activate the calcium/calmodulin-dependent protein kinase II (CaMKII) pathway and enhance cell survival. Overall, the simulation results suggested that the combined use of pharmacotherapy and tDCS/rTMS treatment, based on its impact on microglial function, can potentially bring additive neuroprotective effect and alleviate reperfusion injury for stroke. Another kinetics-based model constructed by Amato and Arnold (2021) studied the dynamic differentiation process of microglial cells (into M1 and M2 subtypes) following ischemic stroke. The major underlying principle of the model is that the M1-type cells were predominantly differentiated from resting microglial cells upon stimulation by pro-inflammatory cytokines, and M2-type cells were generated from resting microglial cells upon encountering anti-inflammatory factors; meanwhile, M1-and M2-type cells are capable of secreting pro- and anti-inflammatory cytokines to facilitate the aforementioned interactions. The authors then simulated the changes in the quantities of M1-and M2-type cells within the first 72 h after stroke. Additionally, sensitivity analysis was performed to identify key parameters that could effectively reduce the differentiation of M1 microglial cells and simultaneously promote the transformation of resting microglia and M1 microglial cells into anti-inflammatory M2 cells. By simulating different neuroprotectant treatments, the authors suggested that early inhibition of M1 activation can lead to a decreased minimum ratio of M1 to M2 microglia and may allow for a larger number of M2 than M1 cells in the long run. In terms of model limitations, the above two studies of microglia signaling and transition are rather more theoretical and can both be further strengthened with more detailed calibration against experimental data measured at the cell level, thereby achieving higher degrees of model confidence and better predictive power in target identification scenarios.

### 2.3 Brain microvascular endothelia cells in stroke

The blood-brain barrier serves as a selective permeability barrier between the central nervous system (CNS) and the circulatory blood system (Daneman and Prat, 2015). Cerebral microvascular endothelial cells are a critical component of the blood-brain barrier. Compared to ordinary endothelial cells, they form tighter capillary endothelium and use intercellular tight junction proteins to bridge the gaps between cells and ensure a controlled, stable permeability of the BBB (Luissint et al., 2012; Kadry et al., 2020). During ischemic stroke, cerebral microvascular endothelial cells experience a decline in cellular activity due to oxidative stress. The resultant production of superoxide can damage the tight junction proteins, leading to increased BBB permeability and may allow toxic substances from the blood to enter the central nervous system and cause severe tissue damage. Therefore, preserving the integrity of the cerebral microvascular endothelial cells post-stroke is crucial for accelerating the repair of damaged brain tissue and slowing the progression of the stroke-related infarction (Lehner et al., 2011; Casas et al., 2019a; Abdulkadir et al., 2020).

To investigate the role of cerebral microvascular endothelial cells in various brain diseases, Gorick et al. (2022) developed a logic-based ODE model capable of depicting the signaling network within human brain microvascular endothelial cells (BMEC) activated by external stimuli such as VEGF, BDNF, and NGF (nerve growth factor). This model primarily characterized cellular behaviors including glucose transport, pro-growth factor signaling, and cell apoptosis, and furthermore through model sensitivity analysis, the authors identified MMP (matrix metalloproteinase) and NFkB as key therapeutic targets in stroke. By simulating certain physiology-based treatment goals in stroke, the authors emphasized the importance of combination treatment as they noted that even a combination of two therapeutic modulations was insufficient to achieve all of the treatment goals. Such a computational platform that comprehensively integrates diverse findings and data on cerebral endothelial cells can provide important insights for the evaluation of new pharmacological intervention strategies in treating ischemic stroke. From the modeling standpoint, we suggest that several other cell functions with essential significance in stroke such as oxidative stress, metabolism and inflammatory factor signaling should also be considered in such large-scale modeling efforts, in order to provide a more comprehensive characterization of BMEC physiology in stroke progression and recovery.

### 2.4 Neurons in stroke

Neurons are the principal functional units of the brain. They play pivotal roles in the reception and conduction of information and are critical to the brain’s optimal operation. These cells are instrumental in various functions such as perception, information processing, motor control, homeostasis, memory, and the regulation of emotions and behavior, enabling our bodies to adapt and respond to diverse environmental stimuli. Following an ischemic stroke, the ensuing inflammatory response, oxidative stress, and the accumulation of metabolic products due to ischemic hypoxia can severely impair the normal functions of neurons, constituting the primary cause of cerebral dysfunction (Baron et al., 2014; Morris et al., 2018; Zhao Y. et al., 2022).

In a recent study conducted by Kratimenos et al. (2022), a combination of experimental and modeling approaches was employed to investigate the molecular mechanisms underlying neural injury induced by hypoxia. Given that hypoxic conditions stimulate neuronal calcium overload which is critical in the induction of cellular apoptosis, the focus was to model the hypoxia-mediated activation of the SRC (SRC proto-oncogene)/calcium/CaMKK2 (calcium/calmodulin-dependent kinase kinase 2) signaling pathway. The authors first simulated the upregulation of BAX (BCL2-associated X) protein expression and the acceleration of neuronal apoptosis induced by hypoxia, and then they used the model to simulate the inhibitory effect on BAX protein expression under conditions where the SRC inhibitor PP2 (pyrazolopyrimidine) was introduced. Furthermore, through global sensitivity analysis, the authors identified the crucial role of NMDAR (N-methyl-d-aspartate receptor) in hypoxia-induced neuronal apoptosis. Adjusting parameters related to NMDAR activation significantly reduced cytoplasmic calcium concentration, consequently lowering BAX protein expression levels. The study revealed that the inhibitory effect is further enhanced when combining NMDAR intervention with PP2. Overall, this ODE-based model could serve as a starting point to embrace more mechanisms of calcium overload in order to systematically characterize excitotoxity and reveal new therapeutic targets. With future model detail additions such as new regulatory mechanisms of calcium influx, mitochondria metabolism and apoptosis, this model-based platform can provide more comprehensive insights in assisting translational investigations of hypoxic brain injury and neuronal signal transduction.

## 3 Modeling ischemic stroke-induced brain damage at the tissue level

Ischemic stroke at the tissue level involves the complex interplay of excitotoxicity, oxidative stress, inflammation, and endothelial damage (Culmsee and Krieglstein, 2007; Amantea et al., 2009). Excitotoxicity, a process involving neurons and astrocytes, is exacerbated under hypoxic conditions due to the excessive release of glutamate by neurons and reduced glutamate absorption by astrocytes. This glutamate surplus overstimulates NMDARs, leading to calcium influx and activation of calcium-dependent death signal proteins, ultimately resulting in neuronal death (Belov Kirdajova et al., 2020; Shen et al., 2022). In the acute phase, oxidative stress will lead to significant production of ROS and cause primary tissue damage. During reoxygenation, peripheral immune cells can also contribute to oxidative stress by enhancing iNOS (inducible nitric oxide synthase) expression and the formation of oxidizing peroxynitrite which would further result in nitrosative stress and cell damage (Crack and Taylor, 2005; Allen and Bayraktutan, 2009). The inflammatory response in stroke is usually a two-step process: the activation of immune cells within the brain, then followed by the recruitment of peripheral immune cells. Key players in this process include microglia, astrocytes, neutrophils, and macrophages, which secrete inflammatory cytokines, chemokines and engage in phagocytosis of dead cells. Under ischemic conditions, DAMPs (damage-associated molecular patterns) will activate microglia, altering their morphology and function to enhance phagocytosis and secretion of various cell factors and chemokines. These cell factors lead to the accumulation of adhesion molecules on the endothelium, paving the way for peripheral immune cells to enter brain tissue (Jin et al., 2010; Jayaraj et al., 2019). The second phase involves the infiltration of peripheral immune cells, primarily neutrophils and monocytes. These mechanisms will compromise the integrity of the blood-brain barrier, as endothelial cells interconnected by tight junction proteins becomes more permeable. This increased permeability allows molecules and ions from reperfused blood, including plasma proteins, salt, and water, to enter the tissue space. After tPA (tissue plasminogen activator) treatment and clot breakdown, restoration of blood flow to ischemic areas, coupled with increased blood pressure, can result in excessive fluid leakage into the tissue space. This fluid accumulation, particularly in cells, can lead to cytotoxic edema and cerebral edema that may escalate intracranial pressure and tissue stress. Furthermore, the unregulated movement of ions due to permeability changes can alter osmotic pressure, exacerbate edema accumulation and cause cellular acidosis. Severe BBB damage can also allow direct blood entry into brain tissue, forming hematomas and exerting further pressure on surrounding brain tissue (Kim et al., 2012; Huang et al., 2021).

To systematically understand this inflammation cascade, a study by Lelekov-Boissard et al. (2009) examines the dynamics of various types of cells post-stroke, including live and dead neurons, microglia cells, neutrophils, and macrophages. The categorization of more specific cell states involves alive/necrotic/apoptotic neurons, along with activated and resting microglia cells. Employing six ordinary differential equations, the model investigates the inflammatory response within the first 24 h following a stroke. The authors used this model to track changes in cell counts, pro-inflammatory factor levels, infiltration rates of neutrophils and macrophages into brain tissue, spatial and temporal activation of microglia cells, and the effects of phagocytosis. Model simulations suggested that the influx of neutrophils and macrophages into brain tissue are not simultaneous, indicating the need to treat these cells as distinct cell populations with different functions. Additionally, the model suggests incorporating spatial diffusion aspects such as the migration of chemotactic cells from blood to brain tissue and the diffusion of cytokines and chemokines, along with other aspects to be refined by future efforts (e.g., protective elements like complement cascades, endothelial growth factors, neurotrophic factors). In a similar attempt, Di Russo et al. (2010) proposed a hybrid ODE-PDE computational model that intricately involves neurons, astrocytes, microglia, neutrophils, macrophages, cytokines, and chemokines. The model incorporates the following pathophysiological processes during an ischemic stroke: neurons and neuroglial cells undergoing necrosis or apoptosis, cellular demise that activates microglia, activated microglia phagocytose dead cells and produce cytokines and chemokines, and macrophages and neutrophils infiltrating the brain tissue. This model employs 13 equations to simulate the alteration in tissue cell counts (particularly apoptotic and necrotic ones), expression of adhesion factors, as well as level of inflammatory mediators and chemokines over the first 72 h post-stroke. The authors simulated how size of the initial infarction can affect the process of inflammation, and the results showed that the severity of inflammation does not correlate linearly with the infarction size. This finding can help determine the optimal timing of using anti-inflammatory drugs during treatment, as the model suggested that the existence of tissue inflammation may be beneficial when the infarction size is small, and the use of anti-inflammatory drugs in small infarctions may actually exacerbate tissue damage in the long run. However, this conclusion may be driven by the biased model inclusion of primarily M1-type microglia and pro-inflammatory cytokines, as the potential anti-inflammatory M2 microglia response which was not described could also have profound impact on the regulation of drug treatment response.

In terms of BBB research, Orlowski et al. (2011) developed a mechanistic model that focuses on three mechanisms: fundamental brain cell metabolism, pH regulation, and increased H^+^ ions due to ATP consumption during a stroke. The metabolism module encompasses energy exchanges between neurons and astrocytes and nutrient transport via capillaries, while pH regulation involves a cellular buffering system with HCO_3_^−,^ (CO_3_)^2-^, and H_2_CO_3_. The model equations for ATP consumption were formulated quantitatively based on Na^+^ concentration. Model simulations revealed a decrease in H^+^ concentration across neurons, astrocytes, and tissues with reduced cerebral blood flow upon stroke occurrence. To further describe in detail the impact of cerebral blood flow on H^+^ diffusion during ischemia and the roles of cellular ion pumps, in a follow-up work, Orlowski et al. (2013) added ion dynamics that were used to simulate cytotoxic edema and analyze dynamic changes in sodium, potassium, calcium, chloride, and bicarbonate ion concentrations. This new hybrid ODE-PDE model elucidates the evolution of spatial dimensions of neurons, astrocytes and the extracellular space during ischemia, focusing particularly on the impact of sodium ion and metabolite diffusion on cellular volume and subsequent tissue volume changes. Still, this model framework of post-stroke pH dynamics could be augmented in future with additional mechanisms (e.g., more details of chloride and hydrogen ion channels) to enhance its accuracy and predictive capability. From another perspective, Mokhtarudin and Payne (2015) developed a PDE-based model to analyze the impact of reperfusion on the disruption of the blood-brain barrier and the collapse of brain microvessels. This model quantitatively used the filtration of capillaries to simulate the formation of vasogenic edema and used morphological changes in microvessels to study their collapse. The simulation results suggested that microvessel collapse would happen most likely under high reperfusion pressure, low blood osmolarity, decreased blood-brain barrier reflection coefficient, and reduced microvessel stiffness. In terms of model limitations, the role of aquaporin-4, which is crucial in blood-brain barrier stabilization and modulation of brain edema, and arguably the effect of oxidative stress-induced damage on BBB should be considered in a future version, as mentioned by the authors.

## 4 Developing in silico clinical trial models for patients

Thrombolytic therapy and mechanical thrombectomy are the principal therapeutic interventions for ischemic stroke. The primary aim of these treatments is to re-establish blood flow by pharmacologically dissolving or surgically removing the vascular occlusion, thereby restoring perfusion and expediting the functional recovery of ischemic tissues (Green and Shuaib, 2006; Diener et al., 2013; Catanese et al., 2017). Currently, recombinant tissue plasminogen activator (rt-PA) is the only thrombolytic agent approved by the FDA for the treatment of ischemic stroke. Its primary mechanism of action involves converting plasminogen to plasmin, which degrades fibrin within thrombi, facilitating thrombolysis and restoring blood supply to the ischemic regions of the brain. However, the therapeutic window for rt-PA is narrow; administration beyond 4.5 h post-onset not only diminishes its efficacy but also increases the risk of hemorrhagic complications (Coutts et al., 2018; Hacke et al., 2018; Katsanos et al., 2021). Mechanical thrombectomy primarily targets large vessel occlusions, such as those in the middle cerebral artery, rapidly restoring blood flow and preventing extensive cerebral ischemia (Guo and Miao, 2021). Both therapeutic approaches carry inherent risks. Thrombolytic therapy in patients with underlying conditions such as hypertension or coagulation disorders can increase the risk of hemorrhage and may also lead to complications such as infections or recurrent strokes. Various external factors can lead to variability in the outcomes of mechanical thrombectomy, including the size and location of the thrombus, the duration of ischemia, and the patient’s specific physiological condition. Not all patients may experience a restoration of normal cerebral function postoperatively (Lambrinos et al., 2016; Jadhav et al., 2021). Assessing a patient’s indications for surgery is a highly challenging task, necessitating a comprehensive analysis of multiple baseline physiological indices (Mokin et al., 2019; Sarraj et al., 2023). Computational systems biology models can significantly aid in this complex task. By constructing virtual patient models using data from prior research, such an approach can support thrombectomy at multiple time points: preoperatively, individualized physiological data can be used to simulate and analyze potential post-surgical risks, aiding in the determination of a patient’s suitability for surgery; intraoperatively, simulations of different thrombectomy strategies can help maximize brain function preservation and increase the survival rate of brain tissue; postoperatively, these models can assist in devising subsequent treatment plans, enhancing the safety of the therapy and accelerating disease recovery (Table 2).

In a recent publication regarding the INSIST project (IN-Silico trials for treatment of acute Ischemic STroke), a systematic approach was reported in terms of constructing virtual patient models for ischemic stroke to enable prediction of therapeutic outcomes for different patients (Konduri et al., 2020). To systematically simulate blood flow, thrombectomy, brain tissue damage and clinical outcome of acute ischemic stroke (AIS) patients, different computational modules were developed and seamlessly connected under the INSIST modeling workflow. Firstly, a virtual cohort was generated using a variety of collected clinical data, including imaging and thrombus characteristics. Secondly, a biophysics-based computational modeling framework was designed and employed to simulate cerebral blood flow, perfusion, thrombosis, and mechanical thrombectomy at the individual patient level. As seen in a proof-of-concept study by Jozsa et al. (2021), an exemplar PDE-based three-compartment porous microcirculation model capable of describing changes in cerebral blood flow perfusion in both healthy individuals and ischemic stroke patients were computationally implemented, which was validated against CT imaging data from both healthy individuals as well as ischemic stroke patients. For thrombectomy, a PDE-based simulation strategy as described by Luraghi et al. (2021b) incorporates a physics-derived cohesive zone model that describes the viscoelastic properties of the thrombus-artery interface to enable accurate predictions of blood flow profiles following thrombectomy in ischemic stroke patients. In addition, such biophysics-driven modules are designed to be integrated with mechanism-based hypoxic tissue injury modules (for example, the hybrid ODE-PDE modeling work by Orlowski et al. (2013) as described in the second section of this review) to depict damage caused by hypoxia and reperfusion to cells and brain tissue, which would allow a more dynamic multiscale reflection of the pathophysiological processes during stroke initiation and progression.

The third step involves establishing connections and associations between physiological outputs generated from the biophysical models with actual clinical endpoints and outcome indicators for every patient. Finally, the aforementioned biophysical modeling-clinical outcome prediction system will be validated against unused clinical trial data to examine its predictability and applicability at the population level for clinical scenarios. As such virtual patient models include comprehensive depictions of the cerebral vasculature and are grounded in hemodynamics and the morphology of blood vessels and incorporate the actual location of occlusions to position virtual thrombi, they can provide high-throughput, comprehensive portrayal of the alterations in global cerebral blood flow that occur during ischemic stroke and upon surgery for every single patient. This can lead to the derivation of virtual clinical indicators, which would have great potential in formulating precise personalized patient treatment plans. The significant translational value of such *in silico* ischemic stroke trial studies following the above four steps proposed by the INSIST project has been first demonstrated in the publications by Miller et al. (2021), Miller et al. (2023). After integrating the various biophysical and physiological modules as described above, the authors, using their computational workflow, generated 500 virtual patients with M1 segment vessel occlusion, based on data of patients enrolled in the MR CLEAN Trial (Jansen et al., 2018). Following simulated endovascular treatment in all virtual patients, the population-level recanalization rate as predicted by the computational platform matched quantitatively with the reported trial results of MR CLEAN. The authors also explored different trial scenarios including thrombectomy in stroke patients with different thrombi fibrin compositions as well as performance comparison of different commercially available stent retrievers, demonstrating that such *in silico* trials can have a great potential to guide ischemic stroke clinical trial design and patient stratification as well as inform medical device developers on the design and performance of new thrombectomy devices.

## 5 Application of omics profiling and network pharmacology in drug target and biomarker discovery for ischemic stroke

Omics research entails a comprehensive analysis of biological molecules through the collective study of various biological datasets (Krassowski et al., 2020). Omics research methodologies encompass genomics, transcriptomics, proteomics, and metabolomics, among others, facilitating an in-depth understanding of molecular mechanisms at various biological levels, disease pathogenesis, and therapeutic targets for drug treatment (Hasin et al., 2017; Rohart et al., 2017; Durufle et al., 2021). Genomic studies focus on studying the entire genome of organisms. By examining the genome of stroke patients, it can identify the genetic variations that most significantly impact ischemic stroke and elucidates the relationship between different gene phenotypes and disease risk (Cheng et al., 2023). Transcriptomic studies primarily investigate the process by which genes are transcribed into mRNA. By analyzing changes in gene expression in patients with ischemic stroke, it identifies key gene modulators critical to the disease’s onset and progression, thereby discovering valuable molecular biomarkers for early diagnosis and prognosis prediction (Appunni et al., 2022; Gallego-Fabrega et al., 2022). Proteomics studies analyze the types, quantities, and functions of different proteins within biological samples. It has significant potential in identifying protein biomarkers in blood for stroke early diagnostics, therapeutic efficacy assessment and drug discovery (Gu et al., 2021; Hochrainer and Yang, 2022; Kalani et al., 2023). Metabolomic studies focus on the types and quantities of metabolites within organisms. By analyzing alterations in metabolic pathways in the brain tissues of ischemic stroke patients, it is possible to identify the most influential metabolic routes and offer new insights for the discovery of therapeutic targets. The metabolites identified can also serve as indicators used for early diagnosis and disease monitoring (Sidorov et al., 2019; Chumachenko et al., 2022; Li et al., 2023).

An exemplar recent multi-omics study by Li et al. (2020) delved into the molecular changes that occur during the progression of stroke and aimed to identify potential markers and drug targets for stroke treatment. The authors used the mice middle carotid artery occlusion (MCAO) model to simulate stroke and performed proteome profiling on cerebral cortex samples at various time points. Proteomic analysis revealed general upregulation of genes associated with immune response and downregulation of genes related to neurodevelopment throughout stroke progression. To further narrow down potential drug targets, the authors then conducted integrated transcriptomic analyses using multiple Gene Expression Omnibus (GEO) datasets for the same purpose and compared the results with findings from the proteomics data, and three proteins (C3, Apoa4, and S100a9) emerged as potential markers or drug targets for stroke as all three displayed significant upregulation in the MCAO model and the GEO datasets. These results again pointed to the involvement of the complement system in stroke progression and suggested that inhibiting C3 activity could be a potential therapeutic approach for mitigating inflammation and reducing brain injury after stroke. For more information on this topic, the review (Montaner et al., 2020) by Montaner et al. has specifically detailed how such omics-driven computational analyses could help to illuminate the intricate molecular landscape driving stroke progression and provide promising new avenues for targeted interventions in ischemic stroke treatment. We have also summarized in Table 3 recent representative omics-based studies with applications in ischemic stroke translational research.

**TABLE 3:** Selected examples of recent omics-based and network pharmacology studies in ischemic stroke translational research. Abbreviations: MMP12—matrix metalloproteinase 12, SCARA5—scavenger receptor class A member 5, GSEA—gene set enrichment analysis, WCGNA—weighted gene co-expression network analysis, PCA—principal component analysis, PPI—protein-protein interaction.

Summary of omics-based study	Applications in stroke translational research	Refs
Proteomic and transcriptomic analyses of mouse MCAO time-course tissue samples using bioinformatics approaches (e.g., GSEA, WGCNA, hierarchical clustering) revealed that C3, Apoa4 and S100a9 as potential drug targets for stroke	Identification of potential drug targets	Li et al. (2020)
Network pharmacology analyses using multi-omics data pointed to new combination treatment strategies in stroke animal models	Identification of potential drug targets and new combination treatment strategies	Casas et al. (2019b)
Proteomic profiling of mouse DH-MCAO brain tissues and bioinformatics analysis (e.g., PCA, hierarchical clustering) revealed a number of differentially regulated proteins post-stroke	Identification of potential inflammation-related drug targets and pharmacodynamic biomarkers	Gu et al. (2021)
Plasma proteomic profiling of ischemic stroke patients suggested that NTproBNP and MMP12 were independently associated with ischemic stroke risk	Identification of potential diagnostic biomarkers	Kalani et al. (2023)
Bioinformatics profiling (e.g., GSEA, PPI) of mRNA and miRNA datasets reveled 6 mRNAs and 2 miRNAs as potential diagnostic markers for ischemic stroke	Identification of potential diagnostic biomarkers	Xie et al. (2020)
Analyses of circulating proteome in ischemic stroke patients revealed 7 potential biomarkers for stroke risk and SCARA5 as a new drug target.	Identification of potential drug targets and diagnostic biomarkers	Chong et al. (2019)
Network pharmacology profiling in combination with *in vitro*/vivo experiments revealed the potential mechanism of action of Huangxiong herbal formula in treating ischemic stroke and pointed to α-Asarone as the major active ingredient	Identification of potential drug mechanism of action	Zhao et al. (2023a)
Network pharmacology profiling in combination with *in vivo* experiments revealed the potential mechanism of action of Shuxuening herbal formula in treating ischemic stroke	Identification of potential drug mechanism of action	Cui et al. (2020)

On the network pharmacology side, Casas et al. (2019b) used a bottom-up “target-to-data” approach and chose NOX4 (NADPH oxidase 4), a potent producer of superoxide following the onset of ischemic stroke, as the primary protein target to study potential combination therapies for stroke. The authors employed network pharmacology approaches for multi-omics datasets and identified four targets—CYBB (cytochrome B-245 beta chain), NOS2 (nitric oxide synthase 2), NOS3 (nitric oxide synthase 3), and NOS1 (nitric oxide synthase 1) that potentially have synergistic interaction with NOX4. Subsequently, NOS family was selected for experimental validation, and the authors showed that *in vitro* cultured brain cells treated with the NOX4 inhibitor (GKT136901) plus the NOS inhibitor (L-NAME) exhibited superior cell survival compared to the control group (single inhibitor) after oxygen-glucose deprivation. This significant combination effect was further demonstrated in tMCAO mice *in vivo*, suggesting that such a network pharmacology and omics-driven approach could be of great value in deciphering multi-target drug discovery and therapy for ischemic stroke.

## 6 Discussion

As reviewed and discussed here, a number of computational models have been developed to describe the multilevel changes that occur during ischemic stroke, encompassing cellular, tissue, and systems-level alterations within the human body. These computational studies have utilized various types of modeling approaches to analyze and predict numerous quantifiable aspects of the disease, such as changes in blood flow, the extent of infarction, radiological alterations, and clinical indicators. However, each modeling approach has its own advantage and limitation. Omics-driven network pharmacology modeling and analysis are capable of handling vast amounts of biological data in a comprehensive and multi-level manner, and thus it can facilitate efficient and cost-effective screening of therapeutic targets compared to traditional biological experiments. However, this sort of modeling analysis demands high quality experimental data, which means incomplete or low-quality data can result in low-accuracy and biased outcomes (Aderem, 2005; Martin et al., 2011; Somvanshi and Venkatesh, 2014; Meyer and Saez-Rodriguez, 2021). Mechanism-based systems biology models often have rich information regarding signaling pathways and cellular crosstalk; still, establishing association between biological markers and clinical endpoints is always a challenge for neurological disorders. Physics-based models can vividly illustrate the physical changes in cerebral blood flow and the alterations in brain electrical signal conduction following an ischemic stroke. However, they rarely account for the related pathophysiological signal transduction or biological regulatory mechanisms. Therefore, integrating multiple modeling approaches (mechanism-based, physics-based, omics-based) in ischemic stroke research can ideally allow for the combination of their strengths and thereby significantly drive model-based translational applications in various aspects including identification of new therapeutic targets, simulation of clinical trials, design of personalized surgical plans, and prediction of stroke recovery. Such a multi-scale modeling strategy has already demonstrated significant potential in various other disease areas (Zhang et al., 2021; Zhao C. et al., 2022; Zhao C. et al., 2023), so we envision that its application in ischemic stroke will greatly facilitate basic research and translational medicine.

Another emerging computation-intensive research direction in ischemic stroke is machine learning, a branch of artificial intelligence designed to analyze large datasets automatically and make data-driven decisions (Jhaveri et al., 2022; Jabeen et al., 2023). In a recent study by Nishio et al. (2020), deep learning algorithms were applied to construct a model that can assist physicians in analyzing clinical CT images from stroke patients. The authors used existing CT imaging data from ischemic stroke patients to train a two-stage deep learning model, and the resulting model demonstrated outstanding assistive capacities in real clinical scenarios in terms of providing fast and accurate diagnosis according to automatic analysis of patient CT images. In another study by Brugnara et al. (2020), a machine learning model was constructed to predict individualized treatment outcomes for ischemic stroke patients. The study incorporated clinical diagnosis and imaging data as well as treatment outcome data (modified Rankin Scale scores) spanning 3 years for 246 ischemic stroke patients. According to their treatment outcomes, patients were categorized into favorable and unfavorable groups, and a machine learning model based on gradient-boosted classifier was used for prediction. Their results show that the trained machine learning model can accurately predict patient outcomes 3 months post-treatment and identify specific patient characteristics that may lead to unfavorable treatment results. In summary, we envision that in future the aforementioned systems-level modeling strategies, particularly when combined with machine learning, will have increasingly significant roles throughout the diagnosis and treatment of ischemic stroke and hold great promise as high-throughput computational tools in the development of new drug targets and therapeutics.
